# Low-Molecular-Weight Bovine Collagen Peptides Reduce Fat Accumulation in *C. elegans* and Ameliorate Obesity-Related Metabolic Dysfunction and Microbiota Diversity in C57BL/6 Male Diet-Induced Obese Mice

**DOI:** 10.3390/ijms26189149

**Published:** 2025-09-19

**Authors:** Miguel López-Yoldi, Paula Aranaz, José I. Riezu-Boj, Itxaso González-Salazar, Jesús M. Izco, José I. Recalde, Carlos J. González-Navarro, Fermín I. Milagro

**Affiliations:** 1 Center for Nutrition Research, University of Navarra, c/Irunlarrea 1, 31008 Pamplona, Spain; mlyoldi@unav.es (M.L.-Y.); paranaz@unav.es (P.A.); jiriezu@unav.es (J.I.R.-B.); cgnavarro@unav.es (C.J.G.-N.); 2 Navarra Institute for Health Research (IdiSNA), 31008 Pamplona, Spain; 3 Viscofan S.A., 31192 Tajonar, Spain; gonzalezi@viscofan.com (I.G.-S.); izcoj@viscofan.com (J.M.I.); recaldei@viscofan.com (J.I.R.); 4 Department of Nutrition, Food Science & Physiology, Faculty of Pharmacy & Nutrition, University of Navarra, 31008 Pamplona, Spain; 5 Centro de Investigación Biomédica en Red de la Fisiopatología de la Obesidad y Nutrición (CIBERObn), Instituto de Salud Carlos III, 28029 Madrid, Spain

**Keywords:** collagen peptides, obesity, fat accumulation, glucose tolerance, dysbiosis

## Abstract

Collagen and its derivatives, including hydrolyzed collagen peptides, have emerged as promising bioactive compounds with potential benefits in obesity and metabolic syndrome prevention and management. This study aimed to evaluate the potential effects of a low-molecular-weight bovine collagen hydrolysate (COLLinstant^®^ LMW) on metabolic health using *Caenorhabditis elegans* and C57BL/6 diet-induced obese mice. In *C. elegans*, C-LMW (2 mg/mL) improved healthspan by significantly reducing fat accumulation (as measured with Nile Red) and reactive oxygen species measured through dihydroethidium, slowing the aging process measured with lipofuscin, and extending the median lifespan of the nematodes. In 32 male 20-week-old diet-induced obese mice, C-LMW supplementation (1 mg/animal/day) for 8 weeks led to a significant reduction in mesenteric, visceral and total adipose tissue (−28% −15% and −18%, respectively; *p* > 0.05), improved glucose tolerance, and partially restored glucose homeostasis, as demonstrated by intraperitoneal glucose tolerance (−26% AUC, *p* < 0.05). Additionally, collagen hydrolysate supplementation led to a significant impact on gut microbiota composition by increasing microbial diversity and modulating beneficial bacterial populations, which may contribute to the observed metabolic improvements. These findings suggest that bovine-derived collagen peptides exert anti-obesogenic and metabolic regulatory effects, supporting their potential as functional dietary ingredients for obesity management.

## 1. Introduction

The global prevalence of obesity has nearly tripled since 1975 and continues to rise at an alarming rate, positioning it as one of the most pressing public health challenges of the 21st century [1]. Often referred to as a modern pandemic, obesity not only threatens individual health but also imposes substantial burdens on healthcare systems worldwide. Consequently, there is a pressing need to intensify public health policies and scientific research aimed at preventing and managing obesity effectively.

Obesity is a multifactorial chronic disease fundamentally characterized by an excessive fat accumulation, which contributes to the pathophysiology of various metabolic disorders, including dyslipidemia, hepatic steatosis, insulin resistance, and type 2 diabetes [2,3]. Despite advances in understanding its underlying mechanisms, current treatment options remain insufficient to fully normalize the biochemical and physiological disturbances associated with obesity.

In recent years, the gut microbiota has emerged as a key modulator of host metabolism and immune function. Dysbiosis, defined as an imbalance in the composition and function of the gut microbiota, has been associated with metabolic endotoxemia and inflammatory signaling pathways that contribute to the pathophysiology of obesity [4,5]. Therefore, elucidating the interplay between the gut microbiota, inflammation, and metabolic regulation is essential for developing novel therapeutic strategies to prevent and treat obesity-related complications.

Dietary proteins play a multifaceted role in human nutrition, exhibiting a wide range of nutritional, functional, and biological properties. Indeed, during digestion or food processing, proteins can release bioactive peptides that exert health-promoting effects, including antihypertensive, antioxidant, immunomodulatory, and appetite-regulating actions [6]. These bioactivities have prompted growing interest in proteins as functional food ingredients, particularly in the context of preventing or managing chronic conditions such as obesity, cardiovascular disease, and type 2 diabetes, and some of the effects of these bioactive compounds can be mediated by the gut microbiota [7]. The nutritional quality of proteins depends on their amino acid composition, structural conformation, and physiological utilization following digestion and absorption [8]. Recent evidence suggests that collagen and its derivatives, including hydrolyzed collagen and bioactive peptides, may play a beneficial role in the prevention and management of obesity and metabolic syndrome. These compounds, often derived from marine or bovine sources, possess not only nutritional value but also biological activity that can influence key metabolic processes. Previous studies in different models have shown that some collagen peptides can enhance satiety, improve lipid metabolism, and reduce body weight and adiposity [9,10,11]. For example, hydroxyproline (an amino acid that is abundant in collagen) has been shown to protect intestinal barrier, mitigates inflammatory responses and enhance antioxidative ability in the gut in pigs [12]. And hydroxyproline-containing peptides have been reported to have antioxidant activity in vitro through their scavenging capacity for hydroxyl and superoxide anion radical [13].

Moreover, collagen-derived peptides have demonstrated antihypertensive, antioxidative, and glucose-lowering effects, which are relevant to the pathophysiology of metabolic syndrome [10]. These properties make collagen an attractive functional ingredient in the development of dietary strategies and functional foods aimed at mitigating obesity-related comorbidities. Given its favorable safety profile and multifaceted metabolic benefits, hydrolyzed collagen supplementation is emerging as a promising adjunct in lifestyle interventions targeting weight control and cardiometabolic health.

The aim of this study is to evaluate the potential effects of a low-molecular-weight bovine collagen hydrolysate (C-LMW) on metabolic health using *Caenorhabditis elegans* and diet-induced obese mice.

## 2. Results

### 2.1. Collagen Hydrolysate C-LMW Improves Healthspan in C. elegans

**Figure 1:**
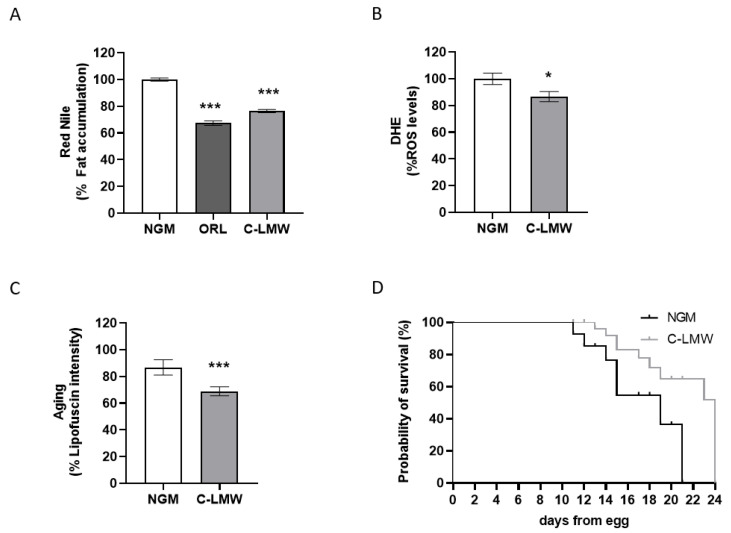
Low-molecular-weight collagen hydrolysate increases *C. elegans* healthspan. (**A**) Nile Red quantification of worms treated with C-LMW or sterile water in NGM. (**B**) Quantification of ROS production (measured by DHE) C-LMW or sterile water-treated worms. (**C**) Quantification of lipofuscin aging pigment in C-LMW and sterile water-treated worms. (**D**) Analysis of lifespan differences between worms treated with C-LMW and those treated with sterile water as a control. Comparisons between two groups were performed using Student’s t-test or the Mann–Whitney U test, after assessing normality with the Kolmogorov–Smirnov and Shapiro–Wilk tests. Comparisons between 3 or more groups were carried out using an ANOVA test followed by Dunnett’s multiple comparisons test when significant differences were obtained. * *p* < 0.05; *** *p* < 0.001 vs. NGM control group.

We initially evaluated the potential of low-molecular-weight collagen hydrolysate to modulate lipid homeostasis in *C. elegans* by assessing their effects on fat accumulation. Interestingly, C-LMW supplementation at the dose of 2 mg/mL resulted in a significant reduction in fat accumulation (≈24%) in *C. elegans* when compared to the untreated control group (Figure 1A). This effect suggests a potential role of C-LMW in modulating lipid metabolism.

C-LMW also led to a significant reduction in ROS levels (≈14%) in *C. elegans* compared to the untreated group (Figure 1B), indicating a potential antioxidant effect. The observed decrease in ROS levels suggests that C-LMW may enhance the oxidative defense system or reduce endogenous ROS production.

Supplementation with C-LMW (2 mg/mL) was also capable of significantly reducing lipofuscin accumulation in *C. elegans* compared to the untreated controls (Figure 1C), suggesting a delay in age-related cellular damage. Lipofuscin, an autofluorescent pigment composed of oxidized proteins and lipids, accumulates progressively with age and serves as a biomarker of oxidative stress and mitochondrial dysfunction [14].

Finally, in line with the previously described results, supplementation with C-LMW at the indicated concentration of 2 mg/mL resulted in a statistically significant increase in the lifespan of *C. elegans* compared to the untreated control group (Figure 1D). The survival curve of the treated group showed a noticeable shift to the right, indicative of extended median (from 19 days in NGM controls to 24 days in the C-LMW-treated worms) and maximum lifespan. The differences observed were statistically significant.

### 2.2. Body Weight Evolution and Final Body Weight in Mice

**Figure 2:**
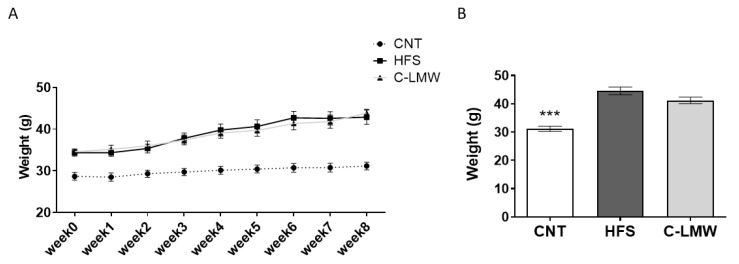
(**A**) Body weight progression over the 8 weeks of the experimental phase in Control, HFS and C-LMW-supplemented mice. (**B**) Final body weight of mice at week 8, after an overnight fasting period. Data are presented as mean ± SEM. Statistical analyses were carried out using an ANOVA test followed by Dunnett’s multiple comparisons test when significant differences were obtained. *** *p* < 0.001 vs. HFS group.

Body weight evolution over the experimental phase (8 weeks) is shown in Figure 2A. Mice fed a high-fat diet (HFS and C-LMW groups) exhibited a continuous and significant increase in body weight throughout the study period compared to the Control group. Starting from similar baseline values, HFS-fed mice exhibited a progressive increase in body weight.

Figure 2B shows the body weight at the end of the study after an overnight fasting period. Consistently with the data shown in Figure 2A, the Control group showed a significantly lower body weight compared to the HFS group. However, no significant differences were observed in body weight or body weight gain between the HFS treated and untreated groups along the experimental phase.

### 2.3. Supplementation with C-LMW Reduces Adiposity in Diet-Induced Obese Mice

The analysis of adipose tissue distribution and organ weights among the experimental groups (CNT, HFS and C-LMW) revealed relevant differences (Table 1). As expected, the control group exhibited significantly lower fat mass across all adipose tissue depots analyzed when compared to the HFS group. Regarding the effects of collagen hydrolysate, it is important to highlight that dietary supplementation with C-LMW resulted in a partial reduction in fat mass compared to the HFS group. Notably, mesenteric and visceral depots, as well as total fat, were significantly reduced. The significant reduction in adipose tissue depots observed in the C-LMW supplemented group compared to the HFS group suggests that collagen hydrolysate may exert anti-obesogenic effects, potentially through modulation of lipid metabolism or adipogenesis.

No significant differences were observed in liver, gastrocnemius muscle weight or spleen weight among the groups (Table 1).

### 2.4. Supplementation with C-LMW Ameliorates Glucose Metabolism in Diet-Induced Obese Mice

**Figure 3:**
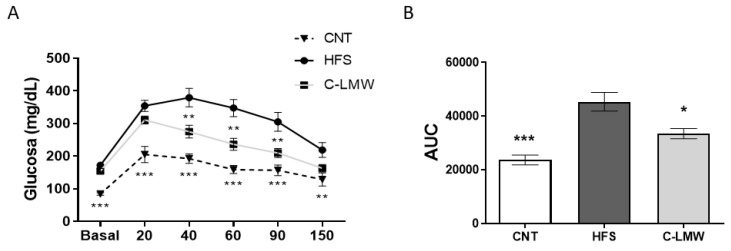
Intraperitoneal Glucose Tolerance Test curve and AUC analysis. (**A**) Intraperitoneal glucose tolerance test (ipGTT) following intraperitoneal glucose administration. (**B**) Total area under the curve (AUC, arbitrary units) of glucose response. Data are expressed as mean ± SEM. *** *p* < 0.001; ** *p* < 0.01 and * *p* < 0.05 vs. HFS group.

An ipGTT was carried one week prior to the euthanasia of mice following an intraperitoneal glucose load. As shown in Figure 3A, intraperitoneal glucose administration induced a rapid increase in blood glucose levels in all groups, with a peak at 20–40 min. The HFS group exhibited significantly impaired glucose clearance, maintaining elevated glucose concentrations throughout the test. In contrast, the group supplemented with C-LMW showed improved glycemic response, with lower glucose levels over time compared to HFS. The Control group maintained the lowest glucose levels across all time points.

In parallel, Figure 3B shows the quantification of the glucose response through area under the curve (AUC) analysis. As expected, the HFS group exhibited a significantly higher AUC compared to the Control group, confirming glucose intolerance of the diet-induce obese model. Moreover, C-LMW showed significantly reduced AUC values compared to HFS, indicating partial restoration of glucose homeostasis following the dietary intervention, indicating that hydrolyzed collagen peptides mitigated the diet-induced impairment in glucose metabolism. These results suggest that dietary supplementation with C-LMW may exert beneficial effects on glucose homeostasis in the context of diet-induced metabolic dysfunction.

To further evaluate the metabolic impact of C-LMW supplementation beyond its observed reduction in total and visceral fat accumulation, we next assessed key biochemical markers related to glucose and lipid metabolism. Table 2 shows the biochemical parameters measured across the experimental groups after the euthanasia of mice, in fasting conditions. As expected, HFS induced marked metabolic alterations characteristic of insulin resistance and dyslipidemia, which are hallmarks of early metabolic syndrome. Interestingly, supplementation with the collagen hydrolysate led to a partial but significant improvement in glycemic parameters, including reduced glucose and insulin levels, and a lower HOMA-IR when compared with the HFS group, suggesting a potential insulin-sensitizing effect.

No statistically significant differences were observed in total cholesterol, HDL cholesterol, cholesterol/HDL ratio, or triglycerides.

Liver enzymes, particularly ALT, were elevated in both the HFS and C-LMW groups relative to the control, indicating ongoing hepatic stress or steatosis, and suggesting limited hepatoprotective effects of the diet supplementation with C-LMW in this model. Overall, these data suggest that the supplementation with collagen hydrolysate C-LMW promotes a partial amelioration of HFS-induced metabolic dysfunctions, particularly regarding glucose homeostasis and insulin sensitivity, though its effects on lipid metabolism appear modest or unclear.

### 2.5. C-LMW Modulates Fecal Microbiota Increasing Alpha Diversity and the Abundance of Beneficial Bacteria in Diet-Induced Obese Mice

**Figure 4:**
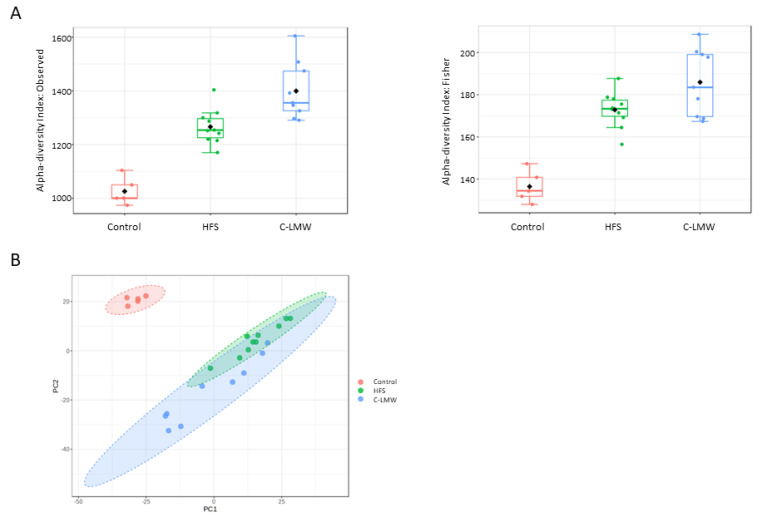
Alpha and beta diversity analyses of bacterial communities across the three experimental groups. (**A**) Boxplot representing alpha diversity (Observed and Fisher Index) in each group: control (CNT), high-fat/sucrose diet (HFS), and collagen hydrolysate-supplemented group (C-LMW). (**B**) Principal Coordinates Analysis (PCoA) based on beta diversity metrics showing clustering of microbial communities according to the treatment group.

We aimed to investigate the potential mechanisms underlying the role of these collagen peptides in preventing obesity and its associated comorbidities. In this sense, Figure 4A shows alpha diversity indices—Observed (left panel) and Fisher’s index (right panel)—across the three experimental groups: Control, HFS, and C-LMW. Both indices reveal statistically significant differences in microbial diversity between groups. Interestingly, there was a significant increase in microbial richness in the C-LMW group compared to HFS (Observed sample diversity index, *p* = 0.006; Fisher diversity index, *p* = 0.048). The Control group showed the lowest diversity.

With regard to beta diversity (Figure 4B), HFS and C-LMW groups showed greater overlap but still formed partially distinct clusters, suggesting that the microbial composition shifted substantially in response to the treatment.

**Figure 5:**
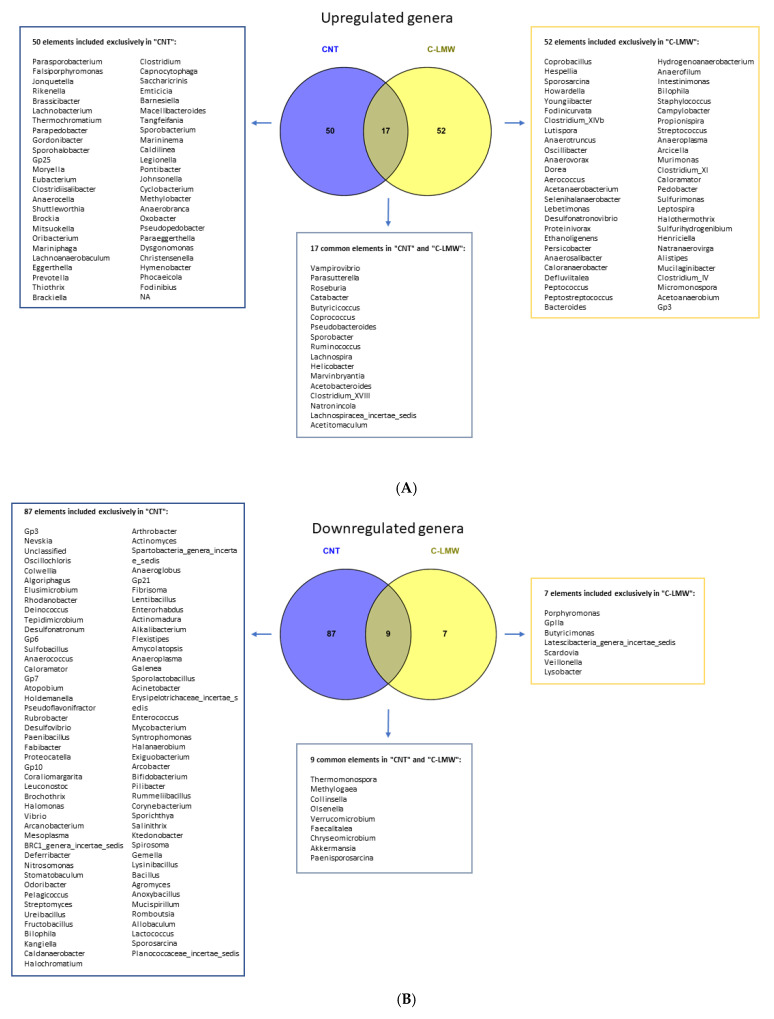
Venn diagram illustrating the genera of bacteria showing increased (**A**) and decreased (**B**) abundance in the Control (blue) and C-LMW (yellow) groups compared with HFS mice.

Once the beneficial effects of C-LMW supplementation on microbiota diversity were confirmed, we aimed to identify genera that showed significant differences in abundance when comparing both the Control group and the C-LMW-supplemented group with the HFS group. In this context, the Venn diagram (Figure 5) illustrates the distribution of microbial genera between the Control, HFS and C-LMW-supplemented groups. A comparative analysis of gut microbiota composition revealed distinct microbial signatures in the Control and C-LMW groups compared to the HFS reference group. A total of 67 genera were significantly upregulated in the Control group, of which 50 were unique to Control, while 69 genera were upregulated in the C-LMW group, with 52 being exclusive to this treatment (Figure 5A). Only 17 genera were commonly upregulated in both groups.

On the other hand, concerning the genera significantly downregulated in response to both Control and C-LMW diet supplementation when compared to the HFS group (Figure 5B), the gut microbiota analysis revealed that 87 genera were exclusively downregulated in Control group, 7 were unique to C-LMW, and 9 genera were commonly suppressed in both groups.

Overall, these data demonstrate that, while both Control and C-LMW groups show an increase/decrease in beneficial bacterial or harmful genera relative to the HFS group, they exhibit distinct microbial profiles, indicating differential mechanisms of microbiota modulation. These changes may underlie the functional metabolic improvements observed in C-LMW-treated groups.

## 3. Discussion

In this study, we first demonstrated that supplementation with low-molecular-weight collagen hydrolysate (C-LMW) at a concentration of 2 mg/mL significantly improves healthspan and promotes longevity in *C. elegans*. One of the key findings was the marked reduction in fat accumulation in the treated group. This observation is consistent with previous studies showing that bioactive peptides derived from collagen exert lipid-lowering and anti-obesity effects in various animal models [15].

Another important aspect of aging is oxidative stress, characterized by excessive production of ROS. Excessive ROS production is a hallmark of oxidative stress and is closely linked to cellular damage and aging processes in *C. elegans* and other organisms [16,17]. Our results indicated that C-LMW supplementation may enhance oxidative stress resistance in *C. elegans*. This is supported by earlier studies showing that collagen-derived peptides can scavenge free radicals and upregulate endogenous antioxidant enzymes, such as superoxide dismutase (SOD) and catalase [18,19]. These findings support the hypothesis that C-LMW supplementation may contribute to improved redox homeostasis, which is critical for longevity and metabolic health in *C. elegans*.

Furthermore, we observed a significant decrease in lipofuscin accumulation, a well-established biomarker of aging in *C. elegans*. The reduction in lipofuscin levels in the C-LMW-treated group suggests that these peptides may attenuate age-related cellular deterioration, potentially through antioxidant and anti-inflammatory mechanisms. Previous studies have shown that bioactive collagen and marine peptides can mitigate oxidative stress and inflammation, both of which contribute to cellular senescence and aging phenotypes in *C. elegans* [20,21,22].

Most notably, C-LMW treatment led to a statistically significant extension of lifespan compared to the untreated control group. These findings suggest that C-LMW may possess bioactive properties capable of modulating longevity pathways in *C. elegans*, potentially through mechanisms involving oxidative stress resistance, mitochondrial function, or insulin signaling, which are commonly implicated in lifespan regulation [23,24]. Similar lifespan-extending effects have been observed in nematodes treated with other marine collagen peptides [18,25]. Further biochemical and genetic analyses are necessary to elucidate the exact physiological mechanisms through which C-LMW exerts its anti-aging effects.

Taken together, our findings highlight the beneficial effects of C-LMW on multiple physiological parameters associated with aging in *C. elegans*, including fat metabolism, oxidative stress, cellular damage, and lifespan. These results established a basis for exploring collagen-derived peptides as potential nutraceuticals for promoting health-promoting benefits in obese mice.

In addition to the beneficial effects observed in *C. elegans*, C-LMW supplementation also demonstrated significant metabolic improvements in diet-induced obese mice. Thus, data of the present study demonstrate that C-LMW supplementation exerts beneficial effects on metabolic parameters in a murine model of diet-induced obesity with no adverse events. This result suggests a potential role of collagen peptides in modulating energy balance or metabolic efficiency and contribute to a healthier metabolic profile. In this context, previous studies by our group and others have reported that collagen may influence body composition by promoting satiety and enhancing lipid metabolism. These effects have been observed in both animal models and human clinical trials [26,27,28,29,30,31,32,33,34,35,36,37,38], supporting the hypothesis that collagen supplementation may contribute to a healthier metabolic profile.

A more pronounced effect of C-LMW was observed in adiposity outcomes. Supplemented mice exhibited significantly reduced total, visceral and mesenteric fat depots compared to HFS-fed animals, indicating a protective metabolic response. In particular, visceral fat is metabolically active and plays a significant role in obesity in mice, contributing to low-grade chronic inflammation and metabolic disturbances [30]. The observed decrease in total and visceral fat depots suggests a protective metabolic effect of the collagen hydrolysate tested, potentially helping to mitigate the complications associated, such as dyslipidemia, hepatic steatosis, and type 2 diabetes. In this context, previous studies have demonstrated that supplementation with collagen peptides or hydrolyzed collagen can reduce adiposity in diet-induced obese mice [27,28,31,32,33]. In relation to mesenteric fat, several studies have associated elevated levels of this depot with both obesity and chronic inflammation [34]. Hence, a potential mechanism of action of the hydrolysate could be the reduction in mesenteric fat, thereby contributing to decrease inflammation.

To further assess the metabolic impact of C-LMW, we evaluated glucose homeostasis. Interestingly, our glucose tolerance test data reveal that C-LMW supplementation also improved glycemic control in the context of diet-induced metabolic dysfunction. In this sense, previous studies have reported that specific bioactive collagen peptides can exert regulatory effects on glucose metabolism. For instance, He et al. [35] demonstrated that dietary supplementation with specific collagen peptides improved type 2 diabetes symptoms in mice. Similarly, a study with a specific collagen hydrolysate demonstrated GLP-1-mediated beneficial effects on oral glucose tolerance in prediabetic mice [36]. Moreover, C-LMW supplementation led to significant improvements in key biochemical parameters of glucose metabolism. Specifically, mice treated with C-LMW exhibited lower fasting glucose, insulin, and HOMA-IR values compared to the HFS group. These results further support the hypothesis that C-LMW exerts insulin-sensitizing effects, potentially contributing to the improved glycemic response observed in the GTT. These findings agree with prior studies which have demonstrated that collagen supplementation can improve glucose metabolism markers in obese mice. For instance, collagen peptides have been shown to alleviate hyperglycemia by modulating insulin resistance, enhancing glucose metabolism, and altering gut microbiota composition in type 2 diabetic mice [37]. Moreover, marine collagen peptides have been reported to improve glucose metabolism and insulin sensitivity in type 2 diabetic rats, likely through the reduction in oxidative stress and inflammation, and upregulation of GLUT4 and PPAR-α expression. Similarly, Ref. [37] demonstrated that collagen peptides derived from *Harpadon nehereus* bones can improve glucose and lipid metabolism in streptozotocin-induced diabetic mice. Altogether, these findings suggest that collagen peptides may be a promising strategy for improving glucose metabolism in obesity-related metabolic disorders.

To further investigate the mechanisms underlying these metabolic improvements, we analyzed the gut microbiota, which plays a crucial role in host metabolism, inflammation, and energy balance. It is well established that microbial alpha diversity, a measure of microbial richness and evenness, is closely associated with metabolic health, and its reduction has been linked to obesity, insulin resistance, and chronic inflammation [38]. Increased diversity is associated with metabolic flexibility and gut homeostasis [39]. Interestingly, C-LMW supplementation significantly increased microbial alpha diversity compared to the HFS group, as shown by higher Observed diversity and Fisher’s diversity index. These findings suggest that collagen hydrolysate supplementation promotes a more diverse microbial community compared to the control and HFS groups. Hence, it can be considered that diet supplementation with C-LMW not only enhances the richness of the gut microbiota but may also directly support fat mass reduction, glucose profile normalization and potentially help to prevent the development of hypercholesterolemia. It is important to note that, although collagen supplementation has been associated with beneficial shifts in gut microbiota composition in obese mouse models, current evidence does not support a significant increase in alpha diversity [31,40].

In addition to richness, microbial composition (beta diversity) was also altered in response to C-LMW treatment. A previous study in diet-induced obese mice supplemented with fish skin-derived collagen peptides also reported a distinct clustering of the microbiota composition in the collagen-treated group, indicating that the supplementation led to a marked shift in gut microbial structure relative to the diet-induced obese group without supplementation [40]. The observed changes in both alpha and beta diversity suggest that C-LMW not only promotes microbial richness but also induces compositional shifts that may contribute to improved metabolic outcomes. Enhanced diversity and beneficial taxa may, in turn, support reductions in fat mass, improvements in glycemic control, and protection against metabolic complications such as hypercholesterolemia.

After confirming the beneficial effects of C-LMW supplementation on gut microbiota, we analyzed bacterial genera that exhibited significant differences in abundance between the HFS group and both the Control and C-LMW-supplemented groups. Genera exclusively upregulated in the C-LMW included some genera that have been associated with diverse metabolic capabilities and, in some cases, potential pathogenicity or dysbiosis. However, it is important to note that the supplementation with collagen peptides also promoted the upregulation of some genera that are well-known for their beneficial effects including *Intestinimonas* or *Bacteroides*, among others. For example, it has been demonstrated that oral administration of *I. butyriciproducens* reduced weight gain, hyperglycemia, adiposity, and inflammation in mice by increasing butyrate production [41]. Moreover, recent articles have shown that administration of some *Bacteroides* strains can protect against weight gain and metabolic dysfunction improving insulin sensitivity, reducing fat mass, and decreasing markers of inflammation [42,43,44]

The 17 genera commonly upregulated in both Control and C-LMW groups, including *Roseburia*, *Ruminococcus* and *Butyricicoccus*, are well-known for their beneficial roles in producing butyrate, an anti-inflammatory short chain fatty acid essential for colonic health [45]. This shared microbial response suggests a partial overlap in the gut microbiota-modulating effects of C-LMM and Control groups, potentially driven by the restoration of metabolic homeostasis and gut microbiota integrity. On the contrary, the C-LMW group showed downregulation of 7 taxa, including *Porphyromonas*. It is important to note that there is growing evidence suggesting that *Porphyromonas* spp., particularly *P. gingivalis*, are associated with obesity and metabolic dysfunction in mice [46,47,48]. Hence, the reduction in this genus of bacteria after C-LMW supplementation in obese mice could represent a positive result for the maintenance of gut balance, potentially contributing to preventing obesity and metabolic dysfunction in mice.

With regard to the 9 genera commonly downregulated in both Control and C-LMW groups, it is important to highlight the decrease in the abundance of *Akkermansia*, *Verrucomicrobium* and *Collinsella*, among others. The decrease in the genus *Akkermansia* is noteworthy, since *Akkermansia muciniphila* is often considered a beneficial bacterium in humans and a decrease in its abundance has been associated with adverse metabolic outcomes in the context of obesity, including increased adiposity, impaired gut barrier function, and insulin resistance. However, it is not the first time that an increased abundance of *Akkermansia muciniphila* has been reported following the consumption of diets high in fat and carbohydrates, including sucrose, maltodextrin, and corn starch [49,50]. This fact can explain the increased abundance of this genus in the HFS group. Finally, a decrease in the abundance of *Collinsella* has been observed in both groups compared to HFS. This genus has been associated with increased gut permeability and pro-inflammatory cytokine profiles, which may contribute to the development and progression of obesity and other metabolic diseases [51,52]. Hence, the observed reduction in *Collinsella* abundance in both Control and C-LMW groups may represent a beneficial outcome compared to diet-induced obese mice.

Although not directly addressed in this study, there are several factors apart from gut microbiota that can explain some of the observed results. For example, collagen hydroxyproline has been reported to protect the intestinal barrier and reduce inflammation [12], and hydroxyproline-containing peptides can have antioxidant activity [13]. On the other hand, we have not observed effects on food intake, although the experimental design was not designed to correctly analyze this parameter. In this context, a recent study described that 15 g/d of bovine collagen peptides following exercise increased GLP-1 and insulin concentrations and reduced ad libitum energy intake at a subsequent meal in physically active females [53]. On the other hand, the effects observed on glucose metabolism can also be independent of the adiposity reduction. In a recent study, Grasset et al. [36] observed that a 5 g dose of the collagen hydrolysate H80 reduced the postprandial glucose response in healthy, normoglycemic and prediabetic persons. In mice, they found that H80 significantly lowered the blood glucose response in lean and prediabetic mice, at a single dose (4 g/kg) much higher than ours. Finally, in a chronic supplementation with the same dose in prediabetic mice, H80 slowed down gastric emptying, and increased plasma active GLP-1 and insulin levels [36].

Overall, our findings indicate that C-LMW supplementation improves multiple aspects of metabolic health in diet-induced obese mice, including reductions in visceral adiposity, improvements in glucose tolerance, and potential hepatoprotection. These results, together with our previous findings in *C. elegans*, support the hypothesis that bioactive bovine collagen peptides may promote systemic metabolic homeostasis and mitigate age- and diet-associated metabolic dysfunction. The difference with other hydrolysates is that, in this occasion, the origin is bovine, while most of the studies have been performed with collagen of marine origin. Also, the methodology applied to hydrolyze the protein is different to previous studies, probably yielding a different pattern of peptides. As a limitation, the quality and quantity of the amino acids can also have an effect on the metabolic outcomes observed in this study. In this aspect, we have not made comparisons with hydrolysates from other collagens or protein sources because it was not the objective of this study. Another limitation is the lack of information about the individual intake of each mouse since they were housed in groups of four; this prevents us from knowing the real intake of peptides of each mouse, and some mice can have eaten more C-LMW than others. In any case, future studies should aim to elucidate the molecular pathways involved, particularly those related to insulin signaling, inflammation, and mitochondrial function, to better understand the therapeutic potential of C-LMW in metabolic diseases.

## 4. Material and Methods

### 4.1. Production Process and Amino Acid Composition of Collagen Peptides from Bovine Sources

The study tested the commercial COLLinstant^®^ LMW (Viscofan DE GmbH, Weinheim, Germany), an oral supplement made from bioactive bovine collagen peptides (types I and III) produced by Viscofan S.A. This hydrolyzed collagen has been previously studied by Carrillo-Norte et al. in human skin health and knee osteoarthritis [54,55]. Supplementary Table S1 shows the amino acid composition of this commercial preparation (in g/100g).

### 4.2. C. elegans Experimental Design

*C. elegans* strain was obtained from the Caenorhabditis Genetics Center (CGC, University of Minnesota, Minneapolis, MN, USA). The N2 Bristol was used as wild-type strain. *C. elegans* were maintained on nematode growth medium (NGM) plates at 20 °C and fed *E. coli* OP50 cultured in LB Broth Lennox (L3022, Sigma-Aldrich, St. Louis, MO, USA) at 37 °C. Experimental assays were conducted in quadruplicate using 6-well plates containing 4 mL of standard NGM.

C-LMW was incorporated into the medium at a concentration of 2 mg/mL and compared with sterile water as negative control (NGM group). Orlistat (1.5 mg/mL, Sigma Aldrich, St. Louis, MO, USA) was incorporated into NGM plates as a positive control for lipid reduction. Plates were allowed to solidify overnight in a dark, dry environment. Afterwards, 150 µL of *E. coli* OP50 overnight culture were spread onto each plate and left to dry at room temperature. Nematodes were synchronized by standard hypochlorite treatment, and the resulting eggs were incubated overnight in M9 buffer at 20 °C. Approximately 200 synchronized L1 larvae were seeded into each well and cultured for two days until they reached the L4 stage, at which point staining and imaging procedures were carried out.

### 4.3. Nile Red and DHE Staining Methods

Both Nile Red and DHE staining methods were carried out as previously described [56]. Nile Red is a fluorescent dye used to detect neutral lipid accumulation. Briefly, L4-stage *C. elegans* previously cultured on NGM under different treatment conditions were collected into 1.5 mL tubes and washed three times with PBST (phosphate-buffered saline containing 0.01% Triton X-100). After that, worms were kept on ice for 15 min and subsequently fixed in 40% isopropanol for 3 min. Staining was performed by adding 150 μL of Nile Red solution (3 μg/mL) to each tube, followed by incubation at room temperature for 30 min with moderate shaking in the dark. After staining, worms were washed again with PBST and mounted onto 2% agarose pads for further fluorescence microscopy analysis.

The fluorescent dye dihydroethidium (DHE; Dihydroethidium BioReagent, ≥95% (HPCE), Sigma-Aldrich, St. Louis, MO, USA) was used to assess reactive oxygen species (ROS) levels in vivo. In brief, 750 synchronized L1-stage *C. elegans* were transferred onto NGM plates supplemented with either sterile water (control NGM group) or C-LMW (2 mg/mL). Upon reaching L4, worms were collected, washed with PBST, and incubated in a 3 μM DHE solution prepared in PBS for 30 min. Following incubation, they were washed again with PBST and mounted on 2% agarose pads containing 1% sodium azide.

### 4.4. Estimation of Aging in C. elegans

Autofluorescence of lipofuscin pigment, an established marker of aging in *C. elegans*, was employed to assess age-related changes. A total of 750 synchronized L1 larvae were transferred to NGM plates supplemented with either sterile water (control NGM group) or C-LMW (2 mg/mL) and allowed to develop to the L4 stage. At that point, worms were collected, washed with PBST, and mounted on 2% agarose pads containing 1% sodium azide.

### 4.5. Lifespan Analysis

Lifespan assays in *C. elegans* were conducted under identical conditions for all treatment groups at 20 °C. Synchronized L1 larvae were transferred to NGM plates supplemented with either sterile water (control NGM) or 2 mg/mL C-LMW for 46 h, allowing development of *C. elegans* to the L4 stage. Each condition was tested in four independent replicates, with 50–65 L4 larvae per replicate. Subsequently, worms were transferred to fresh NGM plates containing 40 µM 5-fluoro-2′-deoxyuridine (FUDR; #856657, Sigma-Aldrich, St. Louis, MO, USA) to prevent progeny production, with no further treatments applied. Survival was assessed daily by gently prodding each worm with a platinum wire; individuals failing to respond were recorded as dead. Monitoring continued until all animals had died.

### 4.6. Image Acquisition and Quantification

For all experimental conditions, approximately 200 nematodes were mounted on microscopy slides following the respective staining protocols. Fluorescent images for Nile Red, DHE and the autofluorescent lipofuscin were acquired using the same imaging settings and procedures described previously [56].

### 4.7. Experimental Design in Mice

All procedures were performed following national and institutional guidelines on laboratory animal care and use and were authorized by the Food Safety and Environmental Health Service of the Government of Navarra, Spain. Ethical approval was granted by the Animal Experimentation Committee of the University of Navarra (protocol number 051-24).

Thirty-two male 20-week-old C57BL/6J male mice (Envigo, Barcelona, Spain) were housed in a clean facility at University of Navarra and maintained in a controlled environment with regulated temperature (21–23 °C), humidity (50% ± 10%), and a 12-h light/dark cycle. Mice were housed in groups of four per cage. The sample size was calculated using weight loss as main variable, based on previous studies from our group in the same mouse strain and diet. Prior to the intervention, all mice underwent a two-week acclimatization period during which they were fed a standard control diet (2014S, Global 14% Protein Rodent Maintenance Diet, Teklad, ENVIGO RMS SPAIN, Barcelona, Spain), with ad libitum access to water and food. After the acclimation period, mice were randomly assigned to either a control (CNT) diet group (n = 8) or a high-fat high-sugar diet group (HFS; D12451, Research Diets, NJ, USA; with 20% of energy corresponding to protein, 35% to carbohydrates and 45% to fat) (n = 24). After 8 weeks of obesity induction through the diet, the mice receiving the HFS were again randomly assigned in a 1:1 ratio to either a HFS group (n = 12) or a HFS group supplemented with collagen-derived peptides (1 mg/mouse/day) (n = 12). The C-LMW peptides were mixed with the HFS until achieving a uniform distribution, and this formulation was prepared every three days. During this dietary supplementation period, animals had free access to food and drink. Cage locations were changed each week by the technical team to minimize confounders. Animals were weighed weekly.

Following eight weeks of dietary supplementation, all animals were euthanized via decapitation in accordance with ethical standards. Trunk blood was collected immediately post-mortem, and serum and plasma were extracted for biochemical evaluation. Organs and tissues, including the liver, spleen, gastrocnemius muscle, and various white adipose tissue depots (retroperitoneal, mesenteric, epididymal, and subcutaneous) were carefully dissected, weighed, and rapidly preserved at −80 °C. We have used the same n for all the analyses, that is the total number of individuals. No animals exceeded the exclusion criteria.

### 4.8. Intraperitoneal Glucose Tolerance Test (ipGTT)

An intraperitoneal glucose tolerance test (ipGTT) was conducted during the 7th week of the dietary supplementation (one week prior to the animals’ euthanasia), as previously described [57]. Briefly, mice were subjected to an 8-h fasting period with free access to water. Then, their body weights were recorded, and each animal received an intraperitoneal injection of D-glucose at a dose of 1 g/kg. Blood glucose levels were monitored at baseline and at 20, 40, 60, 90, and 150 min post-injection by tail vein sampling, using a glucometer and compatible test strips (Optium Plus; Abbott Diabetes Care, Alameda, CA, USA). Glucose concentrations (Gly) were expressed in mmol/L, and the area under the curve (AUC) was determined using the following formula [57]:

AUC_0–180min_ = [20 × (Baseline + 2 × Gly₍_20_₎ + 2 × Gly₍_40_₎ + Gly₍_60_₎)/2] + [30 × (Gly₍_60_₎ + Gly₍_90_₎)/2] + [60 × (Gly₍_90_₎ + Gly₍_150_₎)/2]

### 4.9. Biochemical Analyses

Serum levels of total cholesterol, HDL-cholesterol (HDL-C), the total cholesterol/HDL-C ratio, triglycerides (TG), glucose, aspartate aminotransferase (AST), and alanine aminotransferase (ALT) were measured using the HK-CP kit (ABX Pentra, Montpellier, France) on a Pentra C200 analyzer (HORIBA ABX, Montpellier, France). Insulin concentrations were determined using a specific ELISA kit in accordance with the manufacturer’s instructions (Mercodia AB, Uppsala, Sweden). Insulin resistance (IR) was calculated using the homeostasis model assessment of insulin resistance (HOMA-IR) as follows: [serum glucose (mmol/L) × insulin (mU/L)]/22.5 [58]. Monocyte chemotactic protein-1 (MCP-1) was quantified using specific a commercial ELISA kit (Thermo Fisher Scientific Inc., Waltham, MA, USA and MyBiosource, San Diego, CA, USA).

### 4.10. Fecal Microbiota Analysis

Fecal samples from the different study groups were collected at week eight of dietary supplementation and immediately stored at −80 °C. DNA extraction and bacterial DNA sequencing were conducted at the Genomics Unit of CIMA LAB Diagnostics, Centre for Applied Medical Research (Pamplona, Spain). Double-stranded DNA (dsDNA) was quantified using the Qubit fluorometer (Thermo Fisher Scientific). The V3–V4 hypervariable regions of the 16S rRNA gene were amplified and sequenced following standard Illumina protocols described previously [59,60].

### 4.11. Statistical Analysis

Data are expressed as means ± SEM. Comparisons between two groups were performed using Student’s *t*-test or the Mann–Whitney U test, after assessing normality with the Kolmogorov–Smirnov and Shapiro–Wilk tests. Comparisons between 3 or more groups were carried out using an ANOVA test followed by Dunnett’s multiple comparisons test when significant differences were obtained. For *C. elegans* lifespan assays, statistical significance between collagen-treated and control (NGM) groups was determined using the log-rank (Mantel–Cox) test. Statistical analyses and graph generation were carried out using GraphPad Prism 8.0.2 software (GraphPad Software, Inc., La Jolla, CA, USA). A *p*-value of less than 0.05 was considered statistically significant.

Metagenomic data were analyzed using the MicrobiomeAnalyst platform, https://www.microbiomeanalyst.ca/ (accessed on 15 September 2025) [61]. Alpha diversity was calculated using original/unfiltered data and assessed with a parametric statistical test. Beta diversity was evaluated through Principal Coordinate Analysis (PCoA) based on Bray–Curtis dissimilarity. Statistical significance between groups was tested using PERMANOVA (Feature level). Prior to beta diversity analysis, feature-level data were normalized using the Relative Log Expression (RLE) method.

Statistical differences in microbiota abundances between groups were assessed at genus level using filtered data, retaining features with a minimum of 4 counts in at least 20% of samples and a variance exceeding 10% of the interquartile range. Data were transformed using the Centered Log-Ratio (CLR) method to address compositionality, and group comparisons were performed using *t*-tests or ANOVA.

## 5. Conclusions

The present study demonstrates that C-LMW treatment significantly ameliorates the metabolism of *C. elegans* by reducing fat accumulation, ROS and senescence, and increasing lifespan. Also, it attenuates fat accumulation in mice fed an obesogenic diet, with notable reductions in mesenteric, visceral and total adiposity. These effects were accompanied by favorable changes in biochemical parameters, particularly in glucose tolerance, fasting glucose levels, and insulin sensitivity, suggesting a positive impact of C-LMW on systemic glucose homeostasis. Importantly, these metabolic benefits were accompanied with favorable changes in gut microbiota composition, characterized by increased microbial diversity and modulation of the relative abundance of specific bacterial genera. Altogether, these results support the potential of C-LMW as a promising therapeutic approach for alleviating obesity and its related metabolic disturbances, possibly through mechanisms involving both systemic metabolic regulation and gut microbiota modulation.

## Figures and Tables

**Table 1:** Adipose tissue and organ weights in the three experimental groups at the end of the dietary intervention.

	CNT	HFS	C-LMW
Epididymal fat (g)	0.54 ± 0.09 ***	1.85 ± 0.16	1.90 ± 0.11
Retroperitoneal fat (g)	0.25 ± 0.05 ***	0.86 ± 0.06	0.70 ± 0.09
Subcutaneous fat (g)	0.32 ± 0.05 ***	2.22 ± 0.25	1.70 ± 0.20
Mesenteric fat (g)	0.32 ± 0.06 ***	1.37 ± 0.12	0.99 ± 0.11 *
Visceral fat (g)	1.11 ± 0.19 ***	4.20 ± 0.18	3.59 ± 0.20 *
Total fat (g)	1.43 ± 0.23 ***	6.42 ± 0.34	5.29 ± 0.39 *
Liver (g)	1.10 ± 0.05 **	1.50 ± 0.11	1.24 ± 0.07
Gastrocnemius muscle (g)	0.19 ± 0.03	0.22 ± 0.01	0.23 ± 0.01
Spleen (g)	0.14 ± 0.03	0.14 ± 0.01	0.14 ± 0.01

Values are expressed as mean ± SEM. Visceral fat is referred to the sum of mesenteric, epididymal and retroperitoneal fat depots; Total fat corresponds to the sum of visceral fat and subcutaneous fat. Statistical analyses were performed by Student’s t-test, after assessing normality with the Kolmogorov–Smirnov and Shapiro–Wilk tests. *** *p* < 0.001; ** *p* < 0.01 and * *p* < 0.05 vs. HFS group.

**Table 2:** Biochemical and metabolic parameters in the three experimental groups after dietary intervention.

	CNT	HFS	C-LMW
Glucose (mg/dL)	105.9 ± 14.7 ***	219.5 ± 7.44	190.5 ± 9.2 *
Insulin (µg/L)	0.3 (0.2–0.4) ***	2.8 ± 0.3	1.6 ± 0.3 *
HOMA	2.4 ± 0.7 ***	37.9 ± 4.8	19.0 ± 3.4 **
Total cholesterol (mg/dL)	116.6 ± 3.1 ***	187.8 ± 10.6	160.1 ± 10.2
HDL Cholesterol (mg/dL)	60.7 ± 5.4 *	76.1 ± 3.2	70.28 ± 3.6
Cholesterol/HDL-Cholesterol	2.0 ± 0.1 *	2.5 ± 0.1	2.2 (2.0–2.3)
Triglycerides (mg/dL)	90.7 ± 4.8	91.7 ± 4.9	84.1 ± 6.9
ALT (U/L)	60.1 ± 5.0	91.5 ± 13.1	88.9 ± 11.4
AST (U/L)	372.3 ± 49.3	340.6 ± 32.4	374.4 ± 39.4

Values are expressed as mean ± SEM. Statistical analyses were performed by Student’s t-test or the Mann–Whitney U test, after assessing normality with the Kolmogorov–Smirnov and Shapiro–Wilk tests. *** *p* < 0.001; ** *p* < 0.01 and * *p* < 0.05 vs. HFS group.

## Data Availability

Data are contained within this article. Raw data are available on request from the corresponding author.

## Supplementary Materials

The following supporting information can be downloaded at: https://www.mdpi.com/article/10.3390/ijms26189149/s1.
