# Supplementary material for: Low-Molecular-Weight Bovine Collagen Peptides Reduce Fat Accumulation in C. elegans and Ameliorate Obesity-Related Metabolic Dysfunction and Microbiota Diversity in C57BL/6 Male Diet-Induced Obese Mice
Source: Int J Mol Sci. 2025 Sep 19;26(18):9149. doi: 10.3390/ijms26189149 (PMC12470321; doi:10.3390/ijms26189149)
Supplement: Supplementary file 1 [file ijms-26-09149-s001.zip › ijms-3846712-supplementary.pdf]

Table S1. Amino acid composition of the low-molecular-weight bovine collagen hydrolysate (COLLinstant® LMW).

**Amino acid**	**g/100g of hydrolysate**
Glycine	23.90
Proline	13.51
Hydroxyproline	11.17
Glutamic acid	10.07
Alanine	9.70
Arginine	8.23
Aspartic acid	6.11
Lysine	3.88
Serine	3.02
Leucine	2.91
Valine	2.26
Phenyalanine	1.96
Threonine	1.58
Isoleucine	1.43
Tyrosine	0.72
Histidine	0.65
Methionine	0.64
Ornithine	0.14
Taurine	<0.05
Cysteine	<0.05
